# Spondylodiscite brucellienne et facteurs associés au pronostic : une série de cas à Sétif, Algérie

**DOI:** 10.48327/mtsi.v5i1.2025.563

**Published:** 2025-01-02

**Authors:** Wahiba GUENIFI, Houda BOUKHRISSA, Abdelkader GASMI, Abdelmadjid LACHEHEB

**Affiliations:** Faculté de médecine, Université Ferhat Abbas, Sétif, Algérie; Service des maladies infectieuses, Centre hospitalier universitaire, Saadna Abdennour, Sétif, Algérie

**Keywords:** *Brucella*, Brucellosis, Spondylodiscitis, Epiduritis, Prognosis, Treatment, Setif, Algeria, Maghreb, Northern Africa, *Brucella*, Brucellose, Spondylodiscite, Épidurite, Pronostic, Traitement, Sétif, Algérie, Maghreb, Afrique du Nord

## Abstract

**Introduction et objectifs:**

La spondylodiscite représente une complication sévère et évocatrice de la brucellose. Peu d’études lui ont été consacrées en Algérie bien que l'infection demeure un problème de santé publique. Ce travail vise à rapporter les aspects épidémio-cliniques de la spondylodiscite brucellienne et identifier les complications neurologiques.

**Matériel et méthodes:**

Il s'agit d'une analyse descriptive d'une cohorte de patients adultes atteints de spondylodiscite brucellienne, basée sur les données collectées dans les dossiers de patients recrutés entre janvier 2016 et décembre 2022.

**Résultats:**

Treize femmes et 24 hommes avec un âge moyen de 48 ans ± 15 ans [21 à 71 ans] ont été inclus dans l'étude. Le diagnostic a été posé en moyenne 120 jours ± 100 [30-360] après le début des signes cliniques. Les symptômes fréquemment rapportés étaient : rachialgies (100 %), fièvre (46 %), sueurs (70 %), asthénie (84 %), frissons (22 %) et amaigrissement (27 %). Les complications neurologiques étaient multiples et parfois variées chez le même malade : un cas de paraplégie, trois cas de paraparésie, six cas de troubles sensitifs et un cas de troubles sphinctériens. L'atteinte du rachis lombaire était majoritaire, observée chez 22 patients dont 15 à l’étage L4-L5. L'imagerie a objectivé, outre les lésions disco-vertébrales, 27 cas d’épidurite, 13 cas de compression médullaire, 25 cas de compression radiculaire, en plus des abcès pré ou para vertébraux et du psoas. Deux schémas thérapeutiques ont été utilisés : doxycycline-cotrimoxazole-gentamycine (22 cas) et doxycycline-rifampicine-gentamycine (15 cas). À l'issue du suivi post thérapeutique d'une année, nous avons observé une rechute, une paraparésie séquellaire et des rachialgies séquellaires chez 12 patients.

**Discussion et conclusion:**

Cette étude a permis d'observer des éléments concernant le pronostic. La réalisation précoce d'une imagerie rachidienne est essentielle pour la lutte contre le retard diagnostique trop marqué chez nos patients. La littérature scientifique ne fournit pas de consensus clair sur les complications neurologiques, telles que l'épidurite, ni sur le traitement optimal. Les résultats obtenus dans notre étude peuvent contribuer à l'élaboration d'algorithmes de prise en charge plus personnalisés.

## Abstract (en)

**Introduction and objectives:**

Spondylodiscitis is a serious and suggestive complication of brucellosis. Few studies have been devoted to it in Algeria, although the infection remains a public health problem. The aim of this study was to report the epidemiological and clinical aspects of brucellosis spondylodiscitis and to identify the neurological complications.

**Material and methods:**

This is a descriptive analysis of a cohort of adult patients with *Brucella* spondylodiscitis, based on data collected from patient records recruited between January 2016 and December 2022.

**Results:**

Thirteen females and 24 males with a mean age of 48 ± 15 years [21 to 71 years] were included in the study. Diagnosis was made on average 120 days ± 100 [30-360] after the onset of clinical symptoms. Commonly reported symptoms were: back pain (100%), fever (46%), sweating (70%), asthenia (84%), chills (22%), and weight loss (27%). Neurological complications were numerous and sometimes varied in the same patient: one case of paraplegia, three cases of paraparesis, six cases of sensory disturbances, and one case of sphincter disorders. Involvement of the lumbar spine was predominant, observed in 22 patients, including 15 at the L4-L5 level.

In addition to discovertebral lesions, imaging revealed 27 cases of epiduritis, 13 cases of spinal cord compression, 25 cases of radicular compression, and pre- or para-vertebral and psoas abscesses.

Two treatment regimens were used: doxycycline-cotrimoxazole-gentamycin (22 cases) and doxycycline-rifampicin-gentamycin (15 cases). At the end of the one-year post-therapeutic follow-up, we observed one relapse, sequelae of paraparesis, and sequelae of spinal pain in 12 patients.

**Discussion and conclusion:**

This study has allowed us to observe prognostic elements. Early spinal imaging is essential to combat the excessive delay in diagnosis in our patients. There is no clear consensus in the scientific literature regarding neurological complications, such as epiduritis, and optimal treatment. The results of our study may contribute to the development of more personalized management algorithms.

## Introduction

La brucellose est une zoonose de répartition mondiale. Son incidence annuelle, certainement sous-estimée, varie entre 0,025 et 200 pour 100 000 habitants. Elle est répandue dans la région méditerranéenne, l’Inde, la péninsule arabique, certaines régions de l’Amérique du Sud et Centrale et le Mexique. La brucellose est devenue rare dans les pays développés grâce à la lutte contre la maladie animale et la pasteurisation du lait [20]. Toutefois une recrudescence des cas humains est notée récemment dans certains pays d’Europe suggérant la nécessité d'une vigilance continue [6]. Les bactéries du genre *Brucella* peuvent être responsables chez l'homme d'une maladie sévère et invalidante bien que rarement fatale, ce qui explique leur classification parmi les agents incapacitants. Elles produisent une infection systémique avec des symptômes non spécifiques pouvant évoluer vers des complications touchant différents organes, nécessitant un traitement long parfois complexe. L'atteinte ostéoarticulaire est la complication la plus fréquente et la plus évocatrice de la brucellose. Elle survient chez 10 % à 85 % des patients infectés. Tout le squelette, à la fois les os et les articulations, peut être touché. La spondylodiscite (SPD) est une complication sévère qui peut concerner jusqu’à 54 % des patients. Elle atteint tout le rachis, en particulier lombaire, et peut être uni ou multifocale. L'absence de symptômes spécifiques et l’évolution lente entrainent souvent un retard dans le diagnostic. Le tableau clinique peut simuler une tuberculose vertébrale, à l'origine de l'appellation ancienne de Pott mélitococcique [12, 35].

Le pronostic est étroitement lié à l'installation de complications neurologiques, source d'un handicap moteur parfois irréversible. Il peut s'agir de compressions médullaires ou radiculaires et d’épidurites parfois abcédées. Le traitement comporte des associations antibiotiques diverses et les durées de traitement sont controversées. Cet article vise à présenter une synthèse sur l’épidémiologie, les signes cliniques et les données paracliniques de la SPD brucellienne chez l'adulte et à identifier les complications neurologiques.

## Matériel et méthodes

Il s'agit d'une analyse descriptive rétrospective d'une série de cas de SPD brucellienne de l'adulte colligée entre 2016 et 2022 dans le service des maladies infectieuses du Centre hospitalier universitaire de Sétif, ville semirurale et capitale des hauts plateaux de l’Est algérien. Le diagnostic de SPD était retenu sur l'association d'une symptomatologie clinique évocatrice d'atteinte rachidienne et la présence de lésions vertébrale à l'imagerie. L’étiologie brucellienne était retenue sur l'isolement de *Brucella* spp. dans un prélèvement biologique (hémocultures réalisées systématiquement et mise en culture du pus d'abcès en cas de prélèvement) ou sur une séro-agglutination de Wright positive à une dilution supérieure à 1/80 en présence de signes cliniques et radiologiques évocateurs. Le diagnostic par PCR n’était pas disponible. Les étiologies brucellienne et tuberculeuse ont été recherchées systématiquement en cas de SPD en raison de leur endémicité dans le pays. En plus des données épidémio-cliniques et évolutives, le bilan tuberculeux comprenait une radiographie voire une tomodensitométrie (TDM) thoracique, la recherche du bacille tuberculeux dans les secrétions bronchiques, les urines et les prélèvements de pus, l'intradermoréaction à la tuberculine et l'histologie en cas de biopsie.

Le suivi des patients était quotidien les jours d'hospitalisation, puis tous les quinze jours en consultation pendant la durée du traitement, puis une fois par mois.

L’étude a porté sur les données de routine (épidémiologiques, cliniques, biologiques, radiologiques et thérapeutiques) collectées dans les dossiers de patients par les médecins traitants. L’étude a été faite par le logiciel SPSS version 18 pour Windows.

## Résultats

Au cours de la période d’étude, 331 personnes atteintes de brucellose ont été suivies dans le service, parmi lesquelles 37 patients adultes (13 femmes et 24 hommes) ont été inclus dans l’étude, avec un âge moyen de 48 ans ± 15 ans [21 à 71 ans]. Parmi eux, 25 patients (68 %) étaient âgés de 40 ans et plus. Vingt et un patients (57 %) résidaient dans une zone rurale et 7 d'entre eux (19 %) exerçaient également une profession à risque. Sept autres patients (19 %) ont déclaré consommer des produits laitiers non pasteurisés. Le diabète, principalement de type 2, était une comorbidité fréquente, retrouvée chez 22 patients

(60 %). Parmi eux, six patients présentaient un diabète mal contrôlé.

La spondylodiscite était une localisation secondaire inaugurale chez tous les patients. Le délai entre le début des symptômes et le diagnostic était en moyenne de 120 jours ± 100 jours [30-360]. L’évolution a dépassé 3 mois chez 23 patients. Les rachialgies étaient constantes, associées à une hyperlordose chez 5 patients et à une rectitude rachidienne chez 24 autres. Des complications neurologiques, variées et parfois multiples, ont été observées chez sept patients. Par ailleurs, de multiples manifestations extra-vertébrales ont été identifiées (Tableau I).

**Tableau I:** Manifestations cliniques observées chez les patients

Signes cliniques	N
Rachialgies	37
Fièvre >38°	17
Asthénie	31
Sueurs	26
Frissons	8
Amaigrissement	10
Altération de l’état général	10
Splénomégalie	1
Hépatomégalie	1
Paraplégie	1
Paraparésie	3
Troubles sensitifs : douleurs radiculaires, fourmillements ou picotements	6
Troubles sphinctériens : rétention vésicale	1
Sacro-iliite	7
Atteinte neuro-méningée	2
Surdité	2

Des hémocultures ont isolé des *Brucella* spp. chez 11 malades (30 %). La sérologie de Wright était positive chez tous les malades avec un titre variant de 1/160 à 1/9 920. Ce titre était compris entre 1/160 et 1/320 chez 22 patients (59 %). Cependant, *Brucella* n'a pas été isolée dans le pus des abcès de psoas prélevés chez 6 patients par ponction échoguidée. L’étude histologique de la biopsie osseuse scanno-guidée, pratiquée chez 4 malades, a montré des lésions inflammatoires non spécifiques. La vitesse de sédimentation (VS) était inférieure à 50 mm chez 20 malades (54 %) et entre 50 et 100 mm chez 17 patients (46 %). La protéine C-réactive (CRP) était entre 10 et 50 mg/l chez 33 malades (89 %). Elle n'a dépassé 50 mg/l que dans 4 cas. Dans un seul de ces cas, elle a été supérieure à 100 mg/l. Une hyperleucocytose était notée chez 6 patients (16 %).

Vingt-sept malades ont bénéficié d'une TDM complétée par une imagerie par résonnance magnétique (IRM), 9 patients d'une IRM seule et 1 patient d'une TDM seule. Au plan topographique, 36 patients (97 %) ont présenté une atteinte monofocale tandis qu'un patient a présenté une atteinte bifocale touchant 2 étages lombaires contigus. L'atteinte du rachis lombaire était majoritaire, observée chez 22 patients (59 %), principalement à l’étage L4-L5, tandis que 15 patients avaient une atteinte au niveau du rachis cervical ou dorsal (Tableau II).

**Tableau II:** *Étages vertébraux atteints

Étages	N
C3-C4	1
C4-C5	1
T6-T7	1
T8-T9	2
T11-T12	5
T12-L1	5
L1-L2	1
L2-L3	2
L3-L4	2
L4-L5	15
L5-S1	3

Le total de 38 alors qu'il y a 37 patients est du au fait le fait qu'un malade avait deux lésions lombaires (L3-L4 et L4-L5)

**Figure 1:**
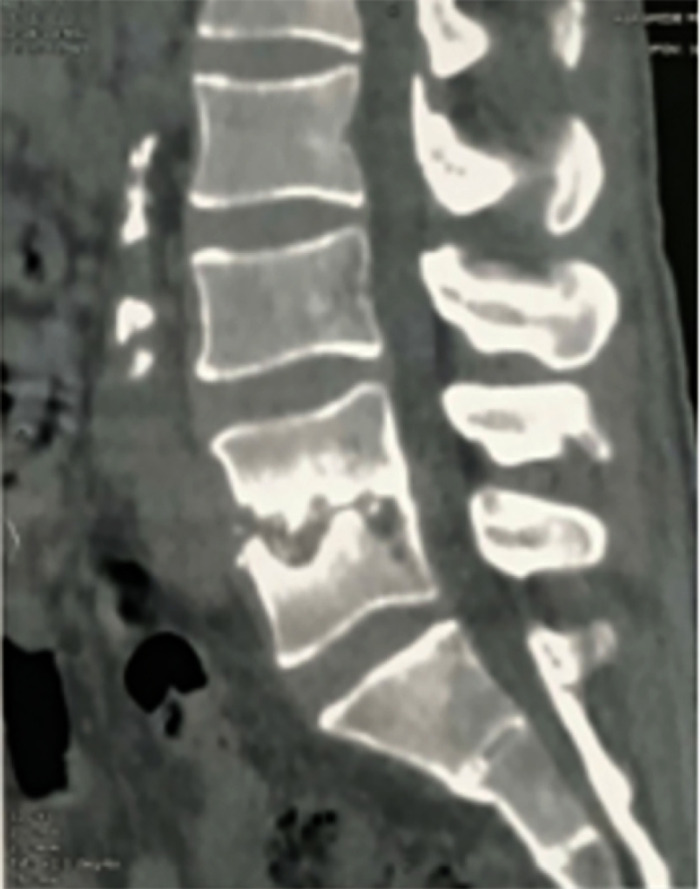
TDM lombaire montrant un pincement discal L4-L5 avec tassement vertébral, érosion et ostéosclérose sous chondrale

L'imagerie a montré des lésions disco-vertébrales isolées chez 5 malades (14 %). Pour les autres, elle a objectivé des lésions constituant des agressions neurologiques (épidurite, abcès épidural, compression médullaire ou radiculaire), des abcès pré ou para vertébraux et des abcès du psoas uni ou bilatéraux (Tableau III). Parmi les lésions disco-vertébrales observées, il y avait deux cas de tassement vertébral (Fig. 1 et 2).

**Tableau III:** Constatations objectivées à l'imagerie du rachis

Lésions retrouvées	N
Atteinte disco-vertébrale isolée	5
Épidurite	27
Abcès épidural	6
Compression médullaire	13
Compression radiculaire	25
Abcès pré-vertébral	11
Abcès para-vertébral	9
Abcès du psoas unilatéral	5
Abcès du psoas bilatéral	6

**Figure 2:**
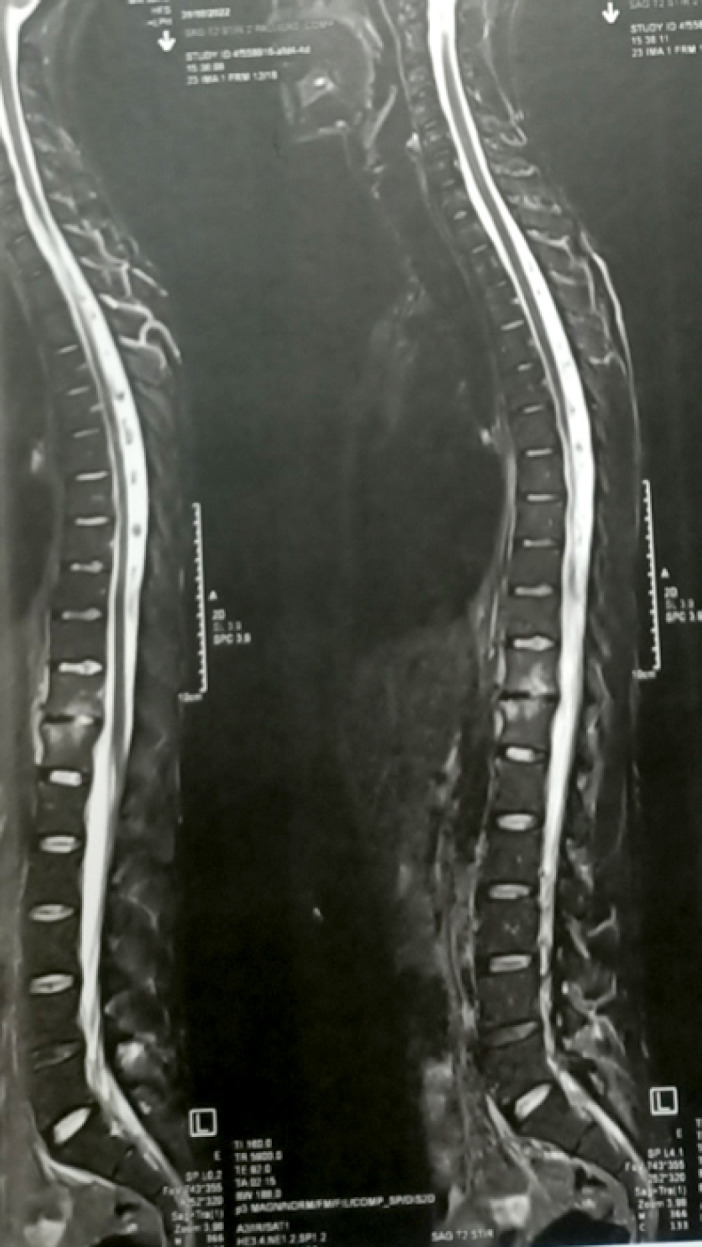
TIRM médullaire montrant un pincement discal D11-D12 avec œdème des plateaux vertébraux

Cinq patients ont été hospitalisés durant une semaine, 17 pendant deux semaines et un patient pendant un mois. Les autres patients n'ont pas été hospitalisés. Deux protocoles antibiotiques ont été utilisés : doxycycline-cotrimoxazole-gentamycine (22 cas, 60 %) et doxycycline-rifampicine-gentamycine (15 cas). La gentamycine a été administrée pendant 15 jours dans les deux cas. La durée totale du traitement par les antibiotiques autres que la gentamycine a varié de 12 à 24 semaines en fonction de l’évolution clinique. Parmi les patients, 24 (65 %) ont reçu une corticothérapie. Quatre patients ont bénéficié d'un drainage radioguidé de l'abcès du psoas. Aucun patient n'a nécessité de recours à la chirurgie. Une immobilisation a été assurée par un corset en résine pour 26 patients et par une ceinture lombaire pour 11 autres. Une rééducation a été mise en place chez 31 patients (84 %).

À l›issue d'un suivi post-thérapeutique d'une année, nous avons observé une rechute survenue deux mois après l›arrêt d›un traitement de 4 mois, une paraparésie séquellaire et des douleurs rachidiennes séquellaires chez 12 patients.

## Discussion

En Algérie, la brucellose est une maladie endémo-épidémique. Son incidence, qui variait entre 20 et 30 pour 100 000 habitants entre 2016 et 2021, a légèrement baissé à 18,2 pour 100 000 habitants en 2021 puis à 17,7 pour 100 000 habitants en 2022. Néanmoins, elle demeure un problème de santé publique important comme en témoigne son classement en tête de liste des zoonoses déclarées en 2022, avec un taux de 54 %, suivie de la leishmaniose cutanée (42 %) [16].

La fréquence des SPD dans la brucellose varie de 2 % à 54 %, selon l'espèce de *Brucella* concernée, les critères de sélection et la population de patients étudiés [36, 37]. Notre travail retrouve 37 cas de SPD, colligés entre 2016 et 2022, parmi les 331 cas de brucellose recensés dans le service (11,2 %) au cours de cette période. Pour une durée identique (2001-2007), un travail réalisé par la même équipe avait rapporté 11 cas de SPD parmi 258 cas de brucellose recensés dans le même service (4,3 %) [14]. Cette augmentation marquée dans la fréquence des cas de SPD nous oblige à réévaluer la place actuelle des formes focalisées au cours de la brucellose à Sétif et dans l'ensemble du pays. Les *Brucella* infectent les os en « ciblant » les ostéoblastes. Elles perturbent leur fonctionnement normal, les poussant à se détruire, à ne plus produire de tissu osseux et à attirer d'autres cellules immunitaires qui aggravent l'inflammation [34]. La prédominance masculine et l’âge moyen dépassant 40 ans dans cette série concordent avec les tendances de la majorité des études publiées qui rapportent que la SPD brucellienne intéresse essentiellement l'homme au-delà de 40 ans. Ces constatations sont également observées au cours des SPD tuberculeuses ou à germes pyogènes [9, 11]. Toutefois, quelques études relèvent une prédominance féminine [23].

Le diabète est retrouvé avec une fréquence élevée (59 %) dépassant largement sa prévalence moyenne dans le pays (14 % en 2018) [25], une constatation dont nous ne pouvons tirer aucune conclusion. Les études publiées ne rapportent pas de fréquence particulière du diabète au cours de la brucellose [33]. Une prévalence élevée du diabète au cours de la brucellose a été constatée durant les années 1950 mais n'a pas été prouvée par les analyses statistiques [22]. Une prédisposition génétique a été rapportée en 2003 par une équipe espagnole qui avait trouvé une association des complications ostéo-articulaires au cours de la brucellose avec le gène HLA-B39 [5].

Le délai diagnostic long (dépassant 3 mois chez 62 % des patients) est lié à une symptomatologie peu bruyante et peu spécifique de l'infection. La brucellose est généralement plus rapidement évoquée en cas de formes aiguës typiques (fièvre sudoro-algique) qu'en cas de formes chroniques, atypiques ou de localisations secondaires. Ces formes chroniques sont attribuées à la capacité de *Brucella* d’échapper à l'inactivation intracellulaire, d'inhiber le TNF α et de moduler la réponse immunitaire. Pour les mêmes raisons, l’état général reste conservé malgré la longue évolution chez la plupart des patients [13, 26]. Les signes généraux (fièvre, sueurs, asthénie) observés avec des fréquences élevées dans notre série contrastent avec la majorité des études qui décrivent des symptômes généraux peu fréquents. Les quelques études qui ont trouvé des résultats identiques aux nôtres correspondent à un interrogatoire minutieux réalisé par des spécialistes en infectiologie [2].

Un taux de positivité à 30 % des hémocultures concorde avec la littérature qui rapporte des taux de positivité de 30 à 70 % dans les formes focalisées [10, 19]. L'espèce de *Brucella* n'est généralement pas identifiée dans notre laboratoire, mais on sait que *B. melitensis*, classique chez les petits ruminants, ovins et caprins, est la souche la plus fréquemment isolée en Algérie [7]. Lorsque la bactérie n'est pas isolée, le diagnostic repose le plus souvent sur le sérodiagnostic de Wright dans les zones d'endémie comme la nôtre. Actuellement, les tests PCR sur le sérum ou des échantillons de tissus, tels que les os, permettent un diagnostic rapide avec des taux de sensibilité et spécificité élevés, mais ils ne sont pas un outil de diagnostic de routine [37].

Le long délai pour parvenir au diagnostic est en grande partie attribuable au retard dans la réalisation de l'imagerie, en particulier de l’IRM du rachis. En raison d'une symptomatologie clinique peu évocatrice de la brucellose, les pathologies mécaniques comme la hernie discale et l'arthrose sont souvent suspectées en premier lieu, conduisant à une prescription empirique d'anti-inflammatoires [17]. Or, l’IRM rachidienne tient un rôle essentiel dans le diagnostic et la prévention des complications des SPD. Elle met en évidence les déformations vertébrales, les extensions épidurales parfois abcédées, les mécanismes des compressions neurologiques et les abcès pré et para-vertébraux [12]. L’IRM peut objectiver une image pathognomonique, le signe de Pedro Pons, où la lésion se présente comme une destruction des vertèbres au coin antérosupérieur accompagnée d'une ostéosclérose proéminente [4, 38]. Certains autres éléments sont évocateurs de l'infection à *Brucella* tels qu'une architecture vertébrale intacte malgré les preuves d'ostéomyélite vertébrale diffuse, associée à une atteinte minimale des tissus mous para-vertébraux [12].

La répartition des atteintes en fonction des étages dans notre série est identique à celle rapportée par la quasi-totalité des séries. Le rachis lombaire est le siège de 69 % des atteintes, suivi du rachis dorsal (19 %) et du rachis cervical (12 %). L'atteinte lombaire se localise préférentiellement au niveau des vertèbres L4-L5, comme observé dans notre étude. Le caractère plurifocal des lésions est observé chez 3 à 14 % des patients et les étages touchés sont rarement non contigus [8, 12, 18, 24, 37]. Si on le compare aux autres étiologies, i) le rachis lombaire est également le plus touché au cours des SPD à germes pyogènes avec prédilection pour les étages L3-L4 et L4-L5 [29]; ii) au cours du Mal de Pott, la localisation dorsale prédomine et la fréquence de l'atteinte multifocale varie de 3 % à 35 % [1, 21, 27, 31]. L'atteinte des tissus mous paravertébraux est minime au cours des SPD brucelliennes (10 à 20 %), plus importante dans les infections à germes pyogènes (40 %) [30] et elle atteint un taux très élevé (70 % à 100 %) dans la tuberculose [9, 11, 21, 27].

Les complications neurologiques de la SPD bru-cellienne, bien que moins fréquentes que dans la tuberculose ou les infections à germes pyogènes, peuvent être sévères. Des cas de paraplégie, bien que rares, ont été rapportés [36]. Une étude comparative a révélé une prévalence de 19 % de déficits neurologiques dans les spondylodiscites à *Brucella*, contre 76 % pour la tuberculose et 61 % pour les infections à germes pyogènes [9]. En cas de brucellose, l'extension épidurale atteint 56 % dans certaines séries (19 % de nos malades) [4]. Elle intéresse essentiellement l’étage cervical [4, 12, 30, 32, 40]. L'incidence de cette complication est nettement supérieure dans la tuberculose (65 à 90 % des cas) [9, 11, 28], justifiant un recours plus fréquent à la chirurgie [4, 36, 41].

Malgré les progrès diagnostiques, le traitement des SPD infectieuses reste complexe faute de consensus thérapeutique et de données probantes issues d'essais cliniques [3, 15]. Les schémas d'antibiothérapie des SPD brucelliennes sont multiples et parfois controversés. Une étude portant sur 293 patients atteints de brucellose spinale, qui a comparé cinq schémas de traitements antibiotiques, n'a pas montré de différence significative en termes de résultats [39]. Une autre étude a rapporté une plus grande efficacité des schémas thérapeutiques comportant un aminoside [35].

## Conclusion

Cette étude a permis de faire ressortir plusieurs éléments pouvant améliorer le pronostic des patients. Ainsi, la réalisation précoce d'une imagerie rachidienne est primordiale en cas de douleurs rachidiennes. Elle pourrait réduire le retard de diagnostic et les complications neurologiques. Bien que l'effectif de notre étude soit limité, la fréquence élevée des complications neurologiques, notamment les épidurites, souligne la nécessité de développer des protocoles de prise en charge plus personnalisés afin d'améliorer le pronostic des patients atteints de SPD brucellienne.

## Source de financement

Ce travail n'a bénéficié d'aucune source de financement.

## Contribution des auteurs et autrices

GUENIFI Wahiba : analyse descriptive des données, rédaction de l'article et approbation de la version finale.

BOUKHRISSA Houda : recueil des données, rédaction de l'article et approbation de la version finale.

GASMI Abdelkader : rédaction de l'article et approbation de la version finale.

LACHEHEB Abdelmadjid : rédaction de l'article et approbation de la version finale.

## Liens d'intérêts

Les auteurs et autrices déclarent ne pas avoir de liens d'intérêts.
