# Supplementary material for: Brucella spondylodiscitis and factors associated with prognosis: a case series from Sétif, Algeria
Source: Med Trop Sante Int. 2025 Jan 2;5(1):mtsi.v5i1.2025.563. [Article in French] doi: 10.48327/mtsi.v5i1.2025.563 (PMC12001995; doi:10.48327/mtsi.v5i1.2025.563)
Supplement: Related texts

# Research article: *Brucella* spondylodiscitis and factors associated with prognosis: a case series from Sétif, Algeria

## Introduction

Brucellosis is a zoonotic disease with a worldwide distribution. Its annual incidence, probably underestimated, varies from 0.025 to 200 per 100,000 inhabitants. It is endemic in the Mediterranean region, India, the Arabian Peninsula, parts of South and Central America, and Mexico. Brucellosis has become rare in developed countries due to animal disease control and milk pasteurization [20]. However, an increase in human cases has recently been reported in some European countries, suggesting the need for continued vigilance [6]. Bacteria of the genus *Brucella* can cause severe and disabling, although rarely fatal, disease in humans, which explains their classification as an incapacitating agent. They cause a systemic infection with non-specific symptoms that can evolve into complications involving multiple organs, requiring long and sometimes complex treatment. Osteoarticular involvement is the most common and suggestive complication of brucellosis. It occurs in 10% to 85% of infected patients. The entire skeleton, both bones and joints, may be affected. Spondylodiscitis (SPD) is a serious complication affecting up to 54% of patients. It affects the entire spine, especially the lumbar spine, and can be uni- or multifocal. The absence of specific symptoms and the slow progression of the disease often delay diagnosis. The clinical picture may mimic spinal tuberculosis, which led to the old name of melitococcal Pott's disease [12, 35].

The prognosis is closely related to the development of neurological complications that may lead to irreversible motor disability. These may include spinal cord or radicular compressions and epiduritis, sometimes with abscesses. Treatment involves a variety of antibiotic combinations, and the duration of treatment is controversial. The aim of this article is to summarize the epidemiology, clinical signs and paraclinical data of *Brucella* SPD in adults and to identify neurological complications.

## Materials and methods

This is a retrospective descriptive analysis of a series of adult *Brucella* SPD cases collected between 2016 and 2022 at the Infectious Diseases Department of the University Hospital of Sétif, the main city of the High Plateau of eastern Algeria but with semi-rural aspects. The diagnosis of SPD was based on the association of clinical symptoms suggestive of spinal involvement and the presence of spinal lesions on imaging. *Brucella* etiology was based on isolation of *Brucella* spp. from biological specimens (blood cultures routinely obtained and culture of abscess pus when specimens were obtained) or on a positive Wright serum agglutination at a dilution greater than 1/80 in the presence of suggestive clinical and radiologic signs. PCR diagnosis was not available. Brucellosis and tuberculosis etiologies were systematically sought in cases of SPD, given their endemicity in the country. In addition to epidemio-clinical and evolutionary data, the tuberculosis work-up included a chest X-ray or computed tomography (CT) scan, a search for tubercle bacilli in bronchial secretions, urine and pus samples, an intradermal tuberculin test, and histology in the case of biopsy. Patients were monitored daily during hospitalization, then biweekly in consultation for the duration of treatment, and then monthly.

The study was based on routine data (epidemiologic, clinical, biological, radiologic and therapeutic) collected from patient records by the treating physicians. The study was conducted using SPSS version 18 for Windows.

## Results

During the study period, 331 patients with brucellosis were monitored in the department, of whom 37 SPD adult patients (13 women and 24 men) were included in the study, with a mean age of 48 ± 15 years [21 to 71 years]. Of these, 25 patients (68%) were 40 years of age or older. Twenty-one patients (57%) lived in a rural area and 7 (19%) had a high-risk occupation. Another seven patients (19%) reported consumption of unpasteurized dairy products. Diabetes, mainly type 2, was a common comorbidity found in 22 patients (60%). Of these, six patients had poorly controlled diabetes.

Spondylodiscitis was an initial secondary localization in all patients. The mean time from symptom onset to diagnosis was 120 days ± 100 days [30-360]. In 23 patients, progression exceeded 3 months. Spinal pain was constant, associated with hyperlordosis in 5 patients and with lordosis in 24 others. Neurological complications, varied and sometimes multiple, were observed in seven patients. Several extravertebral manifestations were also noted (Table I).

**Table I:** Clinical manifestations observed in patients

Clinical signs	N
Back pain	37
Fever > 38°	17
Asthenia	31
Sweating	26
Chills	8
Weight loss	10
Altered general condition	10
Splenomegaly	1
Hepatomegaly	1
Paraplegia	1
Paraparesis	3
Sensory disorders: radicular pain, tingling or pins and needles	6
Sphincter disorders: bladder retention	1
Sacroiliitis	7
Neuro-meningeal involvement	2
Deafness	2

Blood cultures isolated *Brucella* spp. in 11 patients (30%). Wright serology was positive in all patients, with titers ranging from 1/160 to 1/9,920. The titer was between 1/160 and 1/320 in 22 patients (59%). However, *Brucella* was not isolated from the pus of psoas abscesses obtained by ultrasound-guided puncture in 6 patients. Histologic examination of scanno-guided bone biopsies in 4 patients revealed non-specific inflammatory lesions. The erythrocyte sedimentation rate (ESR) was less than 50 mm in 20 patients (54%) and between 50 and 100 mm in 17 patients (46%). C-reactive protein (CRP) was between 10 and 50 mg/L in 33 patients (89%). It exceeded 50 mg/L in only 4 cases. In only one of these cases did it exceed 100 mg/L. Hyperleukocytosis was observed in 6 patients (16%).

Twenty-seven patients underwent CT with magnetic resonance imaging (MRI), 9 patients MRI alone, and 1 patient CT alone. Topographically, 36 patients (97%) presented with monofocal involvement, while 1 patient presented with bifocal involvement at 2 contiguous lumbar levels. Lumbar involvement was predominant, observed in 22 patients (59%), mainly at the L4-L5 level, while 15 patients had cervical or dorsal spinal involvement (Table II).

**Table II:** *Vertebral levels affected

Stages	N
C3-C4	1
C4-C5	1
T6-T7	1
T8-T9	2
T11-T12	5
T12-L1	5
L1-L2	1
L2-L3	2
L3-L4	2
L4-L5	15
L5-S1	3

The total of 38 when there are 37 patients is due to the fact that one patient had two lumbar lesions (L3-L4 and L4-L5).

Imaging revealed isolated disc-vertebral lesions in 5 patients (14%). The others showed lesions constituting neurologic attacks (epiduritis, epidural abscess, spinal cord or radicular compression), pre- or paravertebral abscesses, and unilateral or bilateral psoas abscesses (Table III). Among the disk lesions observed, there were two cases of vertebral compression (Fig. 1 and 2).

**Table III:** Spinal imaging findings

Lesions found	N
Isolated disc involvement	5
Epiduritis	27
Epidural abscess	6
Spinal cord compression	13
Radicular compression	25
Pre-vertebral abscesses	11
Para-vertebral	9
Unilateral psoas abscesses	5
Bilateral psoas abscesses	6

**Figure 1:** Lumbar CT scan showing a pinched L4-L5 disc with vertebral compression, erosion and subchondral osteosclerosis

**Figure 2:** Spinal cord MRI showing a pinched D11-D12 disc with oedema of the vertebral endplates

Five patients were hospitalized for one week, 17 for two weeks and one for one month. The remaining patients were not hospitalized. Two antibiotic protocols were used: doxycycline-cotrimoxazole-gentamycin (22 cases, 60%) and doxycycline-rifampicin-gentamycin (15 cases). Gentamycin was administered for 15 days in both cases. The total duration of treatment with antibiotics other than gentamycin varied from 12 to 24 weeks, depending on the clinical course. Of the patients, 24 (65%) received corticosteroid therapy. Four patients underwent radioguided drainage of the psoas abscess. No patient required surgery. Immobilization was provided by a resin corset for 26 patients and a lumbar belt for 11 others. Rehabilitation was provided for 31 patients (84%). At the end of a one-year post-treatment follow-up, we observed a relapse occurring two months after cessation of a 4-month course of treatment, one sequel paraparesis and sequel spinal pain in 12 patients.

## Discussion

Brucellosis is an endemo-epidemic disease in Algeria. Its incidence, which varied between 20 and 30 per 100,000 inhabitants between 2016 and 2021, has fallen slightly to 18.2 per 100,000 inhabitants in 2021 and then to 17.7 per 100,000 inhabitants in 2022. Nevertheless, it remains a major public health problem, as evidenced by its ranking at the top of the list of zoonoses reported in 2022, with a rate of 54%, followed by cutaneous leishmaniasis (42%) [16].

The frequency of SPD in brucellosis varies from 2% to 54%, depending on the *Brucella* species involved, the selection criteria and the patient population studied [36, 37]. In our study, we found 37 cases of SPD, collected between 2016 and 2022, among the 331 cases of brucellosis recorded in the department during this period (11.2%). During a same period of 6 years (2001-2007), the same team reported 11 cases of SPD among 258 cases of brucellosis identified in the same department (4.3%) [14]. This marked increase in the frequency of SPD cases obliges us to reassess the current role of focal forms in the course of brucellosis in Setif and throughout the country.

*Brucella* infect bone by “targeting” osteoblasts. They disrupt their normal function, causing them to self-destruct, stop producing bone tissue, and attract other immune cells that exacerbate inflammation [34].

The male predominance and mean age of over 40 years in this series are consistent with the trends of most published studies reporting that brucellian SPD predominantly affects men over 40 years of age. These findings are also observed in tuberculous or pyogenic SPD [9, 11]. However, a few studies have shown a predominance of women [23].

Diabetes is found with a high frequency (59%), much higher than the average prevalence in the country (14% in 2018) [25], a finding from which we cannot draw any conclusions. Published studies do not report a particular frequency of diabetes during the course of brucellosis [33]. A high prevalence of diabetes during the course of brucellosis was reported in the 1950s, but not proven by statistical analysis [22]. A genetic predisposition was reported in 2003 by a Spanish team who found an association of osteoarticular complications of brucellosis with the HLA-B39 gene [5].

The long delay in diagnosis (more than 3 months for 62% of patients) is related to the little noisy and unspecific symptomatology of the infection. In general, brucellosis is evoked more rapidly in the typical acute forms (sweating algic fever) than in the chronic, atypical forms or secondary localizations. These chronic forms are attributed to the ability of *Brucella* to evade intracellular inactivation, inhibit TNF α, and modulate the immune response. For the same reasons, the general condition of most patients remains preserved despite the long course of the disease [13, 26]. The general symptoms (fever, sweating, asthenia) observed with high frequency in our series are in contrast to the majority of studies that describe rare general symptoms. The few studies that have found results identical to ours correspond to meticulous questioning by infectious disease specialists [2].

A blood culture positivity rate of 30% is consistent with the literature, which reports positivity rates of 30-70% in focal forms [10, 19]. The species of *Brucella* is generally not identified in our laboratory, but *B. melitensis*, classical in small ruminants, sheep and goats, is known to be the most frequently isolated species in Algeria [7]. When the bacterium is not isolated, in endemic areas such as ours, diagnosis is most often based on Wright's serodiagnosis. Currently, PCR tests on serum or tissue samples such as bone allow rapid diagnosis with high sensitivity and specificity, but are not a routine diagnostic tool [37].

The long delay in diagnosis is largely due to delays in imaging, especially MRI of the spine. Due to a clinical symptomatology that is not very suggestive of brucellosis, mechanical pathologies such as disc herniation and osteoarthritis are often suspected first, leading to empiric prescription of anti-inflammatory drugs [17]. However, spinal MRI plays an essential role in the diagnosis and prevention of complications of SPD. It highlights vertebral deformities, epidural extensions (sometimes abscessed), mechanisms of neurological compression, and pre- and paravertebral abscesses [12]. MRI may show a pathognomonic image, the Pedro Pons sign, in which the lesion presents as destruction of the vertebrae at the anterosuperior corner accompanied by prominent osteosclerosis [4, 38]. Other features suggestive of *Brucella* infection include intact vertebral architecture despite evidence of diffuse vertebral osteomyelitis associated with minimal paravertebral soft tissue involvement [12].

The distribution of involvement by vertebral level in our series is identical to that reported in almost all other series. The lumbar spine accounts for 69% of the injuries, followed by the dorsal spine (19%) and the cervical spine (12%). Lumbar involvement is preferentially localized to the L4-L5 vertebrae, as observed in our study. Plurifocal lesions occur in 3% to 14% of patients, and the affected levels are rarely noncontiguous [8, 12, 18, 24, 37]. Compared to other etiologies, i) the lumbar spine is also the most affected area in pyogenic SPD, with a predilection for the L3-L4 and L4-L5 levels [29]; ii) in Pott's disease, dorsal localization predominates and the frequency of multifocal involvement varies from 3% to 35% [1, 21, 27, 31]. Paravertebral soft tissue involvement is minimal in *Brucella* SPD (10-20%), more significant in pyogenic infections (40%) [30], and very high (70%-100%) in tuberculosis [9, 11, 21, 27]. Neurological complications of *Brucella* SPD, although less frequent than in tuberculosis or pyogenic infections, can be severe. Although rare, paraplegia has been reported [36]. A comparative study found a 19% prevalence of neurological deficits in *Brucella* spondylodiscitis, compared with 76% in tuberculosis SPD and 61% in pyogenic infections [9]. In brucellosis, epidural extension reaches 56% in some series (19% of our patients) [4]. It mainly affects the cervical region [4, 12, 30, 32, 40]. The incidence of this complication is much higher in tuberculosis (65-90% of cases) [9, 11, 28], justifying more frequent surgical intervention [4, 36, 41].

Despite advances in diagnosis, the treatment of infectious SPD remains complex in the absence of therapeutic consensus and evidence from clinical trials [3, 15]. Antibiotic regimens for *Brucella* SPD are multifaceted and sometimes controversial. A study of 293 patients with spinal brucellosis, which compared five antibiotic treatment regimens, showed no significant difference in outcome [39]. Another study reported greater efficacy of treatment regimens including an aminoglycoside [35].

## Conclusion

This study highlighted several factors that could improve *Brucella* SPD prognosis. Early spinal imaging is essential in cases of spinal pain. It could reduce the delay in diagnosis and neurological complications.

Although the number of patients in our study is limited, the high frequency of neurological complications, particularly epiduritis, highlights the need to develop more personalized management protocols to improve the prognosis of patients with *Brucella* SPD.

## Funding

No funding was provided for this work.

## Authors’ contributions

GUENIFI Wahiba: descriptive analysis of data, drafting of article and approval of final version. BOUKHRISSA Houda: data collection, writing of the article and approval of the final version. GASMI Abdelkader: drafting of article and approval of final version.

LACHEHEB Abdelmadjid: drafting of article and approval of final version.

## Conflict of interest

The authors declare no conflict of interests.
